# Regulating the spatial distribution of metal nanoparticles within metal-organic frameworks to enhance catalytic efficiency

**DOI:** 10.1038/ncomms14429

**Published:** 2017-02-14

**Authors:** Qiu Yang, Wenxian Liu, Bingqing Wang, Weina Zhang, Xiaoqiao Zeng, Cong Zhang, Yongji Qin, Xiaoming Sun, Tianpin Wu, Junfeng Liu, Fengwei Huo, Jun Lu

**Affiliations:** 1 State Key Laboratory of Chemical Resource Engineering, Beijing University of Chemical Technology, Beijing 100029, China; 2 Key Laboratory of Flexible Electronics (KLOFE) & Institute of Advanced Materials (IAM), Jiangsu National Synergetic Innovation Center for Advanced Materials (SICAM), Nanjing Tech University (NanjingTech), 30 South Puzhu Road, Nanjing 211816, China; 3 Chemical Sciences and Engineering Division, Argonne National Laboratory, 9700S. Cass Ave, Argonne, Illinois 60439, USA; 4 X-Ray Science Division, Argonne National Laboratory, 9700S. Cass Ave, Argonne, Illinois 60439, USA

## Abstract

Composites incorporating metal nanoparticles (MNPs) within metal-organic frameworks (MOFs) have broad applications in many fields. However, the controlled spatial distribution of the MNPs within MOFs remains a challenge for addressing key issues in catalysis, for example, the efficiency of catalysts due to the limitation of molecular diffusion within MOF channels. Here we report a facile strategy that enables MNPs to be encapsulated into MOFs with controllable spatial localization by using metal oxide both as support to load MNPs and as a sacrificial template to grow MOFs. This strategy is versatile to a variety of MNPs and MOF crystals. By localizing the encapsulated MNPs closer to the surface of MOFs, the resultant MNPs@MOF composites not only exhibit effective selectivity derived from MOF cavities, but also enhanced catalytic activity due to the spatial regulation of MNPs as close as possible to the MOF surface.

## Abstract (web summary)

Composites incorporating metallic nanoparticles into metal-organic frameworks are potentially useful but controlling the nanoparticle spatial distribution is challenging. Here, the authors report a strategy that accomplishes this by using a metal oxide as both support and sacrificial template.

Metal-organic frameworks (MOFs), also known as porous coordination polymers, are a class of microporous materials constructed from metal nodes and organic linkers, and have been a major focus of nanoscience over the past decades^1^,^2^,^3^,^4^,^5^. They possess high surface area and uniform cavities, as well as structural and functional tunability, making them promising for a variety of applications^6^, including gas storage^7^,^8^, chemical separation^9^,^10^,^11^,^12^, catalysis^13^,^14^,^15^,^16^,^17^, sensing^18^,^19^ and drug delivery^20^. They can also act as unique host matrices and offer a platform for loading functional species to develop new types of MOF-based composites with novel chemical and physical properties^21^,^22^,^23^,^24^,^25^,^26^,^27^,^28^,^29^,^30^,^31^. In particular, the incorporation of metal nanoparticles (MNPs) in MOFs has been rapidly growing in the field of heterogeneous catalysis^32^,^33^,^34^,^35^,^36^,^37^,^38^,^39^,^40^,^41^,^42^,^43^. Due to the molecular sieving effects from the framework material, the obtained MNPs@MOF composites exhibited excellent size- and shape-selective catalytic properties^44^,^45^,^46^,^47^,^48^. For example, Kuo *et al*.^44^ reported a general strategy to synthesize nanocrystal@ZIF-8 yolk-shell nanostructures for selective heterogeneous catalysis. We have developed a strategy for the encapsulation of noble-metal nanoparticles in MOFs, which exhibited excellent shape selectivity in olefin hydrogenation^45^. Very recently, Li *et al*.^46^ synthesized a Pt–Ni framework within MOF composites for efficient and selective production of imines due to the hydrogen enrichment and molecular sieve effect of the MOF shell. However, despite the excellent catalytic selectivity of MNPs@MOF composites, for most of the research, including our previous results^23^, an unfortunate trade-off is the poor reaction efficiency due to the low diffusion rate of molecules in the narrow channel of the MOF shell. Constructing MNPs@MOF composites with molecular-size selectivity as well as satisfactory efficiency presents a major challenge in spite of the need in selective catalysis.

Pioneering attempts have demonstrated that constructing porous or hollow MOF structures around MNPs is an effective way to improve the catalytic activities of MNPs@MOF composites^35^,^36^,^44^. For example, MNPs@MOF hollow nanocages^35^,^36^,^47^ and MNPs@ZIF-8 yolk−shell nanostructures^44^ were reported to show enhanced catalytic activities, benefiting from the cavity between the MNPs and the MOF shell. In our previous work, we also developed an NP-template strategy to prepare mesoporous MOFs-Pt hybrid materials that display high catalytic activity originating from the mesopores^41^. However, current methods either only succeed in a few examples and are not applicable to various MNPs@MOF composites, or require expensive and tedious processes.

The critical bottleneck to catalysis with MNPs@MOFs lies in the diffusion of reactants when they pass through the long narrow path of the MOF layer to the active sites of the MNPs. If the MNPs are encapsulated near the MOF surface as close as possible, the distance of the reactants from the surface to the active sites within MOFs can be minimized, and thus, higher catalytic efficiency is expected. Achieving this structure requires a fine regulation of the spatial distribution of MNPs within the MOF layer, which had been a major challenge. Herein, we report a facile strategy of regulating the spatial localization of encapsulated MNPs in a MOF layer through transformation of metal oxides to MOFs and simultaneous encapsulation of the MNPs. The spatial localization of MNPs can be regulated, so that they are either kept at the interface between the MOF crystal and the metal oxide, or are moved far away from the metal oxide template (close to the MOF surface) by optimization of the crystallization behaviour of the MOF under different concentrations of organic ligands. We found that the catalytic efficiency of the as-prepared MNPs@MOF composites was significantly enhanced by control of the MNPs as close as possible to the MOF surface, mainly due to the shortened distance of the reactants from the surface of the MOF to the active sites of MNPs encapsulated in MOF. Meanwhile, the size- and shape-selective behaviour that originates from the microporous nature of the MOF component was maintained. In addition, this strategy is general and versatile, and can be extended to other MNPs@MOFs, which can bring more opportunities for optimizing catalysts with high reactivity and selectivity.

## Results

### Encapsulation of MNPs in MOFs with spatial regulation

**Figure 1:**
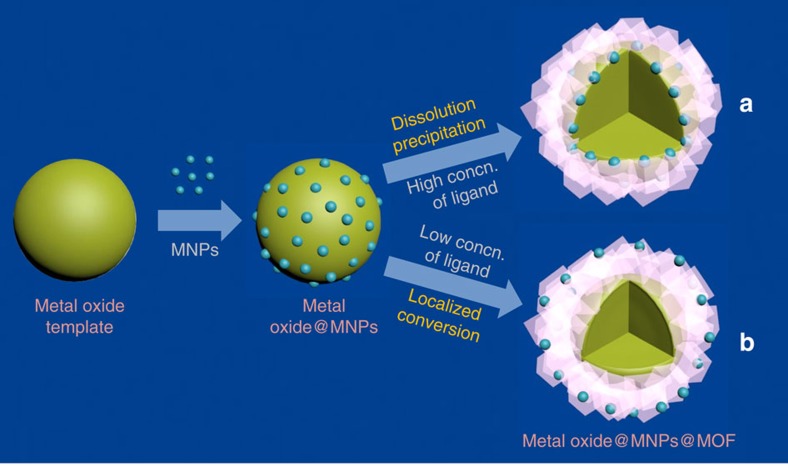
Spatial localization regulation of MNPs within MOF crystals via template-sacrifice method. Through surface modification with surfactant PVP, MNPs of various sizes, shapes and compositions are loaded on the surface of metal oxide, and by reacting the metal oxide/MNPs template with organic ligands, MNPs can be encapsulated in MOF crystals. The spatial distribution of incorporated MNPs within MOF crystals can be controlled by tuning the crystallization behaviour of MOFs under (**a**) high and (**b**) low concentration of the organic ligands.

The controllable encapsulation of MNPs into MOFs with location regulation is based on the sacrificial-template synthetic method^49^,^50^. The MNPs were first loaded onto the shaped metal oxide nanostructures, which were subsequently transformed into MOF crystals by the oxides reacting with organic ligands. The MNPs were encapsulated into MOF crystals during the transformation step (Fig. 1). The spatial localization of the MNPs in the MOF layers could be regulated by tuning the crystallization behaviour of the MOFs under different concentrations of the organic ligands. When a high concentration of ligands was used, the nucleation and growth of MOFs followed a ‘dissolution-precipitation mechanism,' and the MNPs were fixed at the interface between the oxides and MOF layers (Fig. 1a). However, at low concentration of ligands, the transformation of metal oxides to MOFs followed a ‘localized conversion mechanism,' and thereby the MNPs were immobilized at a location much closer to the surface of the MOF (Fig. 1b).

**Figure 2:**
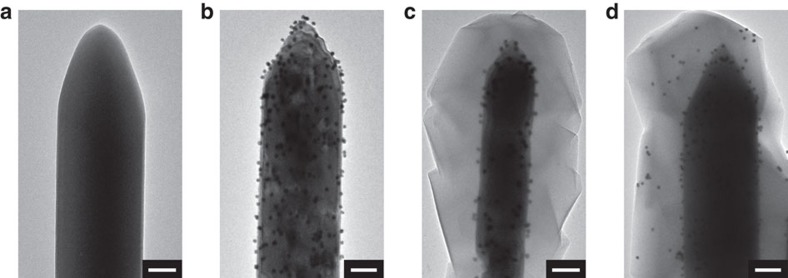
The encapsulation of Au NPs into ZIF-8 with spatial regulation. TEM images of (**a**) ZnO NRs, (**b**) ZnO NRs@Au and (**c**,**d**) ZnO NRs@Au@ZIF-8 composites with Au NPs at different locations: (**c**) inside and (**d**) near to the surface of ZIF-8. Scale bar, 100 nm.

The controllable encapsulation procedure was demonstrated by a series of proof-of-concept experiments. First, ZnO nanorods (NRs) (Fig. 2a) were used as a sacrificial template to prepare Au@ZIF-8 composites. In a typical experiment, ZnO@Au composites (10 mg, Fig. 2b), prepared by loading 13 nm Au NPs (Supplementary Fig. 1a) on ZnO NRs, were added to a dimethylformamide (DMF)/H_2_O (16 ml, 3:1 v/v) solution of 2-methylimidazole (Hmim), followed by keeping the mixture at 70 °C for 12 h. The obtained ZnO NR@Au@ZIF-8 composites were pink, while the supernatant was transparent and colourless, which implied that essentially all of the Au NPs were incorporated in the composite after the 12 h of reaction. The localization regulation of the encapsulated Au NPs was realized by tuning the concentration of the organic ligand (Hmim) at the beginning of the reaction (Fig. 2c,d). The samples synthesized under different ligand concentrations consisted of isolated ZnO NR@Au@ZIF-8 composites with a core-shell structure.

Well-dispersed Au NPs were fully encapsulated in the MOF layers, and no Au NPs were observed on the outside surface of the ZIF-8. When the Hmim concentration was high (125 mM, 2 mmol) in the above reaction system, the Au NPs were fixed at the interface between the ZnO NRs and ZIF-8 (Fig. 2c). However, at a low Hmim concentration (40 mM, 0.64 mmol), a high proportion of the Au NPs was found to be immobilized much close to the surface of the ZIF-8 (Fig. 2d). Powder X-ray diffraction (XRD) measurements confirmed the formation of MOF crystals (Supplementary Fig. 2a). Both samples showed distinct diffraction peaks that originate from ZIF-8 with a cubic space group (I3 m)^51^, accompanied by those assigned to wurtzite-type ZnO (JCPDS no. 36-1451). Peaks associated with Au NPs were too weak to be observed, presumably because of their low concentrations and/or small sizes.

**Figure 3:**
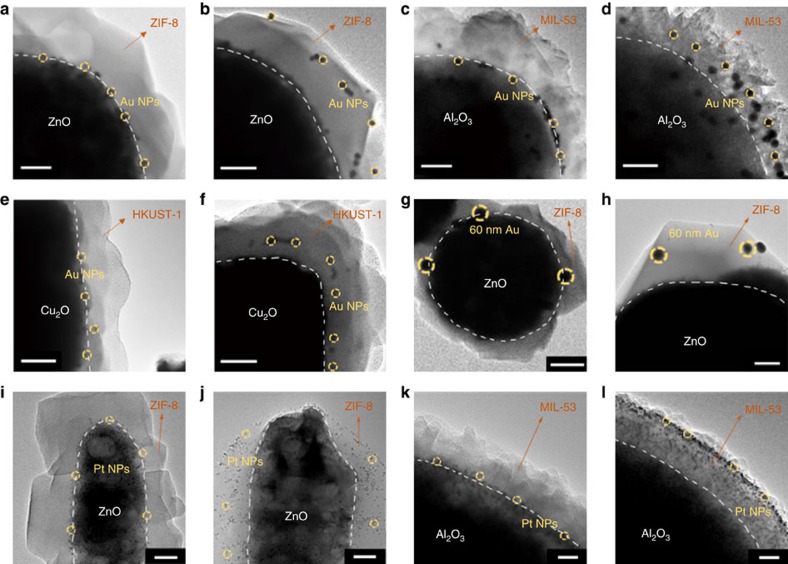
The controllable encapsulation of various MNPs in different MOFs with corresponding metal oxides as templates. TEM images of 13 nm Au NPs@MOF composites obtained under two ligand concentrations: (**a**,**b**) ZIF-8 with ZnO spheres as template, (**c**,**d**) MIL-53 with Al_2_O_3_ spheres as template and (**e**,**f**) HKUST-1 with Cu_2_O cubes as template. TEM images of various types of MNPs@MOF composites: (**g**,**h**) 60 nm Au NPs in ZIF-8 with ZnO spheres as template, (**i**,**j**) 3.3 nm Pt NPs in ZIF-8 with ZnO nanorods as template and (**k**,**l**) 3.3 nm Pt NPs in MIL-53 with Al_2_O_3_ spheres as template. Scale bar, 200 nm (**a**–**h**), 50 nm (**i**,**j**) and 20 nm (**k**,**l**), respectively. In pairs of images, the higher ligand concentration appears on left.

Great versatility of this controllable encapsulation method was found in various MNPs and MOFs with their corresponding metal oxides as templates. First, we verified the method by encapsulating 13 nm Au NPs with different MOFs by changing the sacrificial metal oxides. Specifically, ZnO spheres (∼1 μm), Al_2_O_3_ spheres (∼0.5 μm) and Cu_2_O cubes (∼0.7 μm) were chosen as templates (Supplementary Fig. 1) to encapsulate Au NPs into different MOFs (ZIF-8, MIL-53 and HKUST-1) under two ligand concentrations. At high ligand concentration, the Au NPs were encapsulated into the MOFs at the surface of the oxide cores (Fig. 3a,c and e), while at low ligand concentration, the Au NPs were driven away from the oxide cores to the surface of the MOFs (Fig. 3b,d and f).

The encapsulation process was also adapted to MNPs of 60 nm Au and 3.3 nm Pt (Fig. 3g–l). For instance, 60 nm Au NPs (Supplementary Fig. 1b) were encapsulated in ZIF-8 with spatial localization regulation using ZnO spheres as the template (Fig. 3g, h). Furthermore, 3.3 nm Pt NPs, which have high catalytic activities in a wide range of reactions, were also tested. As shown in Fig. 3i–l and Supplementary Fig. 3, when 3.3 nm Pt NPs were loaded on ZnO NRs, Al_2_O_3_ and Fe_3_O_4_ spheres, similar results were obtained. And it is worth noting that there is almost no loss of the MNPs after capsulation by monitoring the UV–vis absorption of the solution (Supplementary Fig. 4). For all cases, the XRD patterns (Supplementary Fig. 2b–d) confirmed the formation of MOF structures from the corresponding metal oxides. The above results demonstrate that the location of encapsulated MNPs within MOF crystals can be well regulated by tuning the concentration of organic ligands in the transformation process of MOFs.

### Mechanism of localization regulation of MNPs within MOFs

Two mechanisms are proposed for the phase transformations in the MNPs@MOF composite with position-dependent nucleation rate. One is the ‘dissolution-precipitation mechanism'^52^, which is generally accepted in the sacrificial-template synthetic strategy of MOF nanostructures^49^,^50^. This mechanism posits that the dissolution of the parent oxides into metal ions in the fluid takes place before the nucleation and growth processes of MOFs. The other mechanism assumes that the transformation of insoluble oxide/hydroxide into MOFs is strongly localized inside the oxide/hydroxide precursor accompanied by recrystallization^53^. The formation of MOFs can be attributed to a template-localized acid–base interaction between the ligands and the oxides, and the non-soluble nature of the precursor (oxides) and the product (MOFs) makes it possible for the whole process to take place without any release of metal ions into the solution. This mechanism is called the ‘localized conversion mechanism'.

**Figure 4:**
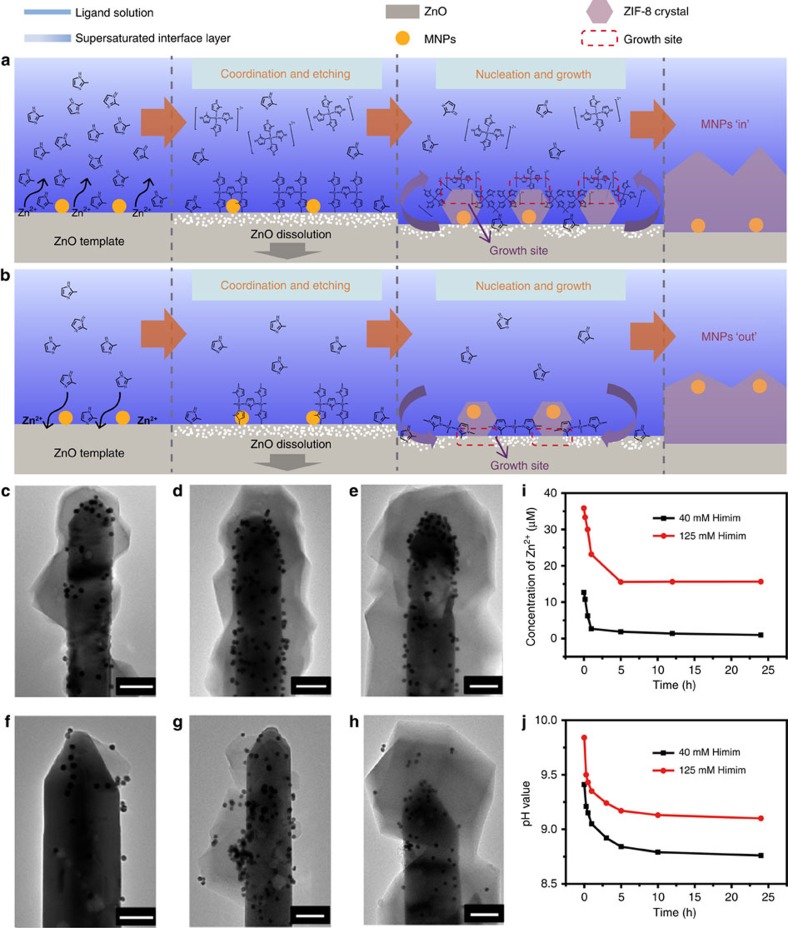
Mechanism and time-dependent experiments of localization regulation of MNPs within ZIF-8. (**a**,**b**) Schematic view of two mechanisms for the encapsulation of MNPs into ZIF-8 with position regulation; (**c**–**h**) TEM images of the product synthesized with high Hmim concentration at the reaction times of (**c**) 0.5 h, (**d**) 1 h and (**e**) 5 h and with low Hmim concentration at the reaction times of (**f**) 0.5 h, (**g**) 1 h and (**h**) 5 h. (**i**) Concentration of Zn^2+^ versus reaction time and (**j**) pH versus reaction time of the ZnO@Au@ZIF-8 at low (black line) and high (red line) concentration of Hmim, respectively. Scale bar, 100 nm.

Based on our experimental results and the above transformation mechanisms, we proposed a mechanism to understand the location regulation of encapsulated MNPs in the MOF layer. The ZnO NRs@Au@ZIF-8 composite was taken as an example, as schematically illustrated in Fig. 4. After the addition of the aqueous suspension of ZnO NRs to the DMF solution of Hmim, two main reactions occurred:


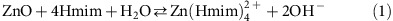



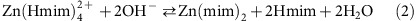


The total reaction could be represented as follow:


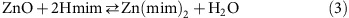


The conversion of ZnO into ZIF-8 at high concentration of Hmim followed the ‘dissolution-precipitation mechanism' (Fig. 4a). A large number of zinc cations at the ZnO surface were coordinated by Hmim, and the Zn(Hmim)_4_^2+^ cations were rapidly released to the reaction solution (equation 1). At the increase of the Zn(Hmim)_4_^2+^ concentration, ZIF-8 (Zn(mim)_2_) nucleated from the oversaturated solution and deposited onto the surface of the templates (equation 2). With continued reaction time, the ZIF-8 crystals grew and connected to each other to form a multi-crystalline layer. In this case, the growth interface of the ZIF-8 crystal is mainly at the outside of the ZIF layer, which leaves MNPs at the initial surface of the metal oxides (fixed at the interface between the ZnO and ZIF-8). On the contrary, for low Hmim concentration, the Zn(Hmim)_4_^2+^ concentration in the solution could not reach the nucleation concentration of ZIF-8. Therefore, equation 2 cannot occur in this condition, and the conversion process is prone to take place on the surface of the ZnO by a direct heterogeneous reaction (equation 3), which can be understood by the ‘localized conversion mechanism' (Fig. 4b). In this case, the corresponding growth interface occurs between ZnO and ZIF-8. Therefore, the growth of ZIF-8 pushes the Au NPs away from their initial position (close to the surface of the composite).

The above two growth processes were verified by time-dependent experiments (Fig. 4c–h and Supplementary Fig. 5). For each case, transmission electron microscopy (TEM) images and XRD patterns were obtained at 0.5, 1 and 5 h. At high initial Hmim concentration (125 mM), we observed that the Au NPs were fixed on the interface between the ZIF-8 and ZnO throughout the process (Fig. 4c–e). In the first 0.5 h, isolated ZIF-8 crystals (∼50 nm) with lower contrast emerged on the ZnO NR surface. Subsequently, those ZIF-8 NPs grew and formed a continuous layer on the ZnO NR surface. At 5 h, the thickness of the ZIF-8 layer gradually increased and reached ∼180 nm. At 12 h, no obvious change was observed.

For the ZnO@Au@ZIF-8 synthesized with low Hmim concentration (40 mM), we found that the Au NPs did not stay at the interface of the ZIF-8 and ZnO, but moved out to the composite surface as the nucleation and growth of ZIF-8 occurred (Fig. 4f,g). The Zn(Hmim)_4_^2+^ concentration monitored by inductively coupled plasma spectroscopy (ICP) also confirmed the differences between the two processes.

As shown in Fig. 4i, the initial Zn(Hmim)_4_^2+^ concentration was 36 μM when the ZnO templates were added into the Hmim solution (125 mM) and heated to 70 °C. This concentration quickly decreased within 1 h, indicating the rapid consumption of Zn(Hmim)_4_^2+^ in the early stages. The decrease in the Zn(Hmim)_4_^2+^ concentration slowed at about 1 h, and remained at about 16 μM after 5 h, indicating that the reactions (Eqs. 1 and 2) had reached equilibrium. The initial Zn(Hmim)_4_^2+^ concentration for a small amount of Hmim (40 mM) was only 13 μM, which is much lower than the initial ligand concentration, and even lower than the equilibrium concentration (16 μM) after 24 h for the high Hmim concentration. The Zn(Hmim)_4_^2+^ concentration in the solution decreased dramatically to a negligible value within 1 h (∼2.5 μM). This process was considered as nucleation of ZIF-8 crystals from the solution, which were deposited onto the surface of the templates and had initially encapsulated the MNPs in the MOF layer. Afterwards, the Zn(Hmim)_4_^2+^ concentration remained low (1∼2 μM), indicating that the whole process takes place without any release of metal ions into the solution. However, as the reaction time increased, localized conversion of ZnO to ZIF-8 occurred, as indicated by the TEM images in Fig. 4g,h. The ZIF-8 crystals grew and formed a continuous layer on the surface of the ZnO NRs, which can be attributed to an acid–base interaction between the zinc oxide and Hmim. In this process, most of the Au NPs were pushed out and stayed close to the ZIF-8 surface. As shown in Fig. 4j, the pH values were also tracked during the reaction, and were found to gradually decrease as the concentration of Zn(Hmim)_4_^2+^ decreased, a trend that is in accordance with equation (2).

**Figure 5:**
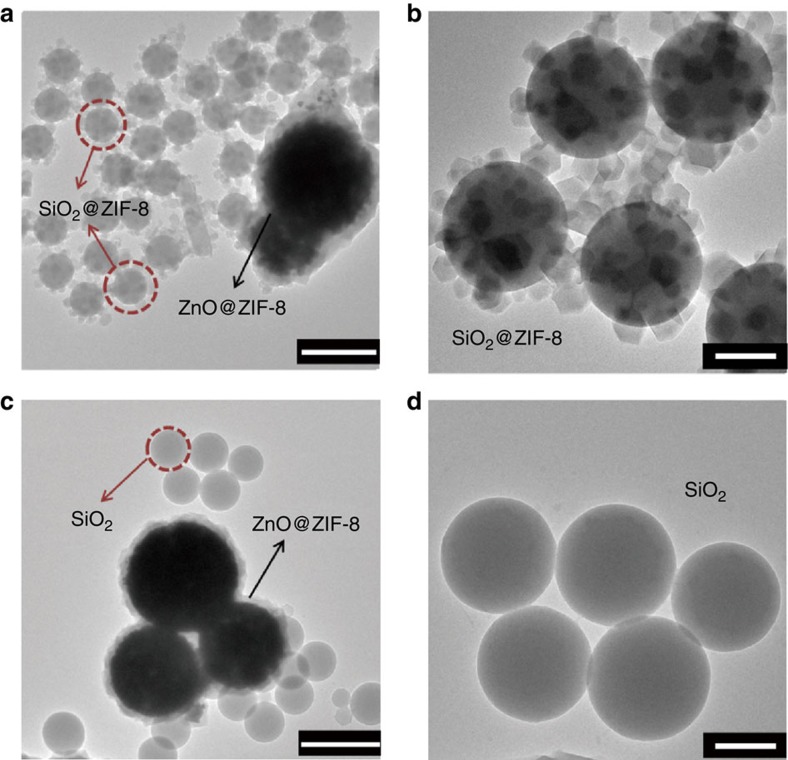
Synthesis of ZnO@ZIF-8 with extra SiO_2_ under different concentration of Hmim. TEM images of the products under (**a**, **b**) high concentration and (**c**, **d**) low concentration of Hmim. Micrographs **b** and **d** show the difference of the surface of SiO_2_ in the two cases. (**b**) The conversion of ZnO into ZIF-8 crystals is followed by the ‘dissolution-precipitation mechanism' at high ligand concentration, resulting in ZIF-8 crystals that are nucleated onto SiO_2_ surfaces. (**d**) The conversion of ZnO into ZIF-8 crystals is followed by the ‘localized conversion mechanism' at low ligand concentration; thus, ZIF-8 crystals would nucleate only onto the surfaces of ZnO. Scale bar, 1 μm (**a**,**c**) and 200 nm (**b**,**d**).

The proposed mechanism for high ligand concentration is that the conversion of metal oxides into MOF crystals is followed by the ‘dissolution-precipitation mechanism', leading to nucleation of the MOF crystals onto the metal oxide and Hmim surfaces. At low ligand concentration, the conversion of metal oxides into MOF crystals is followed by the ‘localized conversion mechanism', leading to nucleation of MOF crystals only onto the metal oxide surfaces. To further validate the above mechanism, SiO_2_ nanospheres (∼300 nm, Supplementary Fig. 6) were added to the system of ZnO spheres and Hmim at similar reaction condition. As expected, at the high Hmim concentration, ZIF-8 nanocrystals grew on the surface of both ZnO and SiO_2_ spheres (Fig. 5a,b), indicating that a large number of Zn^2+^ cations in the solution makes it easy to form ZIF-8 nanocrystals based on the dissolution-precipitation mechanism. At the low Hmim concentration, ZIF-8 nanocrystals were only found to grow on ZnO spheres, indicating that the Zn^2+^ concentration is too low to form ZIF-8 nanocrystals, and the ZIF-8 could only form around the surface of ZnO based on the localized conversion mechanism (Fig. 5c,d). These results are consistent with our proposed mechanism: the high Hmim concentration caused a larger amount of Zn^2+^ in the solution, making the growth of ZIF-8 happen by deposition from the solution, while the low Hmim concentration just made that happen on the interface between the ZnO template and ZIF-8.

### Catalytic activities of MNPs@MOF composites

**Figure 6:**
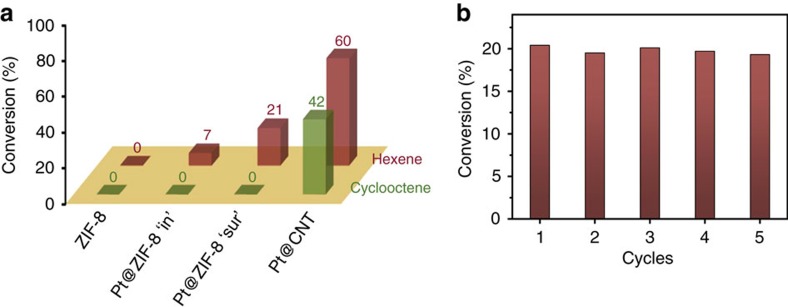
Catalytic properties of ZnO@Pt@ZIF-8 composites for olefin hydrogenation. (**a**) Size-selective hydrogenation of n-hexene and cyclooctene catalysed by ZnO@Pt@ZIF-8 composite (3.3 nm Pt NPs with content of 0.9%); (**b**) the reusability of Pt@ZIF-8-‘sur' as catalysts for the hydrogenation of hexene for five consecutive runs. Reaction conditions: 0.01 g of catalyst, 0.1 ml of substrate, 3 ml of ethyl acetate, 1 atm of hydrogen, 35 °C, reaction time of 12 h. Pure ZIF-8 and Pt@CNT were used as controls.

As mentioned above, the spatial location of MNPs can be controlled near the MOF surface, which will undoubtedly provide fast diffusion in heterogeneous catalysis without the loss of selectivity. The accessibility of embedded 3.3 nm Pt NPs in ZIF-8 with different locations (Fig. 3i,j) was probed by examining the liquid-phase hydrogenation of n-hexene (molecular width of 1.7 Å) versus cyclooctene (molecular width of 5.5 Å). As shown in Fig. 6a, the introduction of the MOF layer around Pt NPs imparts to the composites of obvious size and shape selectivity of the reactants. The Pt@ZIF-8 composites showed catalytic hydrogenation activity to linear n-hexene, but no activity to cyclooctene, owing to the sieving effect from pore restriction of the MOF matrix (small pore apertures of 3.4 Å). Moreover, the Pt@ZIF-8 composites with Pt NPs at different locations exhibited different hydrogenation activity to n-hexene. When encapsulating Pt NPs in the inner part of the MOF layers, the composites (Pt@ZIF-8-‘in') attain 7% conversion of n-hexene hydrogenation. In contrast, the sample with Pt NPs located near the surface of the MOF layers (Pt@ZIF-8-‘sur') showed much higher activity for hydrogenation of n-hexene (conversion of 21%). It is noteworthy that the active sites of Pt@ZIF-8-‘in' and Pt@ZIF-8-‘sur', which were determined by CO chemisorption, are almost the same (120.0 and 117.8 μmol g^−1^_metal_, respectively). Thus, the higher catalytic activity of Pt@ZIF-8-‘sur' compared with Pt@ZIF-8-‘in' can be attributed to the shortened distance of reactant diffusion through narrow channel from the surface to the active sites within MOFs. The catalytic conversions and turnover frequency values were summarized in Supplementary Table 1.

For comparison, Pt@carbon nanotube (CNT) and ZnO@ZIF-8 nanocomposites were also tested for olefin hydrogenation. As shown in Fig. 6a, the ZnO@ZIF-8 exhibited no catalytic activity with respect to the hydrogenation reaction of olefins, which indicates that the catalytic activity of Pt@ZIF-8 composites arises from Pt NPs embedded in ZIF-8. By contrast, the Pt/CNT composite exhibited higher hydrogenation activity but no selectivity for either hexane or cyclooctene. In short, by regulating the MNPs near the surface of the MOF, we not only can achieve high catalytic selectivity, but also significantly higher catalytic efficiency. In addition, the recyclability of the Pt@ZIF-8-‘sur' catalyst was examined. As shown in Fig. 6b, there is no significant loss of activity within five successive catalytic cycles. TEM image and XRD pattern of the used catalyst do not show obvious changes compared with those of the fresh catalyst (Supplementary Fig. 7) as well, indicating the long-term stability of the catalyst.

## Discussion

We have demonstrated an effective strategy to controllably immobilize various MNPs into MOF materials. The location of the MNPs in MOFs can be regulated by optimization of the crystallization behaviour of MOFs under different concentrations of organic ligands and by use of metal oxide with loaded MNPs as sacrificial templates to provide metal ions that grow MOF crystals. This strategy is applicable to a broad range of MOFs and MNPs, and allows the incorporation of multiple non-agglomerated MNPs within the MOFs. Furthermore, the MNPs@MOF composites exhibit a better selective catalysis, which benefits the molecular sieving behaviour of MOFs, together with the high catalytic activity of isolated MNPs due to the improved mass transport.

## Methods

### Materials and measurements

Commercial reagents were purchased from Sigma-Aldrich (ACS grade) and used as received unless otherwise noted. Powder XRD patterns were recorded with a Bruker AXS D8 Advance diffractometer by using nickel-filtered Cu-Kα radiation (*λ*=1.5406 Å). Scanning electron microscope images were taken by a JEOL JSM-7600 field-emission scanning electron microscope with an accelerating voltage of 5 kV. TEM images were taken by a JEOL JEM 2100 TEM at an accelerating voltage of 200 kV. ICP spectroscopy was conducted on a Dual-view Optima 5300 DV ICP-OEM system. The pH value of the reaction system was monitored by Mettler Toledo FE-20. The numbers of active sites on the surface of Pt were determined from CO chemisorption using an automated catalyst characterization system (Autochem II 2920) from Micromeritics Instrument Corporation equipped with a thermal conductivity detector. The samples were treated at 423 K for 60 min and then cooled to room temperature in a He flow of 50 ml min^−1^. The CO chemisorption was measured at 293 K by introducing pulses of 5% CO–He flow (50 ml min^−1^) until adsorption saturation. Stoichiometric factor (Pt:CO molar ratio in the chemisorption) is taken as 1.

### Controllable encapsulation of MNPs in MOFs

Metal oxide@MNPs@MOF composites were synthesized by the sacrificial-template method using metal oxide@MNPs composites as templates. At high ligand concentration, MNPs were fixed on the interface between the metal oxide and MOF, while at low ligand concentration, MNPs were moved out and located near the surface of the MOF shell. Detailed methods for the synthesis of MNPs, metal oxide templates and metal oxide@MNPs@MOF composites are presented in the Supplementary Methods. Here we take encapsulation of 13 nm Au NPs in ZIF-8 as an example to describe the preparation method. In a typical experiment, ZnO NRs@Au (∼10 mg) with a certain amount of Hmim (2 mmol or 0.64 mmol) was added to a glass bottle containing a mixed solvent of DMF/H_2_O (16 ml, 3:1 v/v). After reacting the mixture at 70 °C for 12 h, the products were washed by ethanol three times, and ZnO NRs@Au@ZIF-8 composites with core-shell structure were obtained.

### Preparation of SiO_2_@ZIF-8 composites

SiO_2_ nanospheres, ZnO spheres and a certain amount of Hmim (2 mmol or 0.64 mmol) were added to a glass bottle containing a mixed solvent of DMF/H_2_O (16 ml, 3:1 v/v). After reacting the mixture at 70 °C for 12 h, the products were washed by ethanol three times.

### Preparation of catalysts

The ZnO NRs@3.3 nm Pt@ZIF-8 catalysts were prepared by the above encapsulation method. The Pt content of 0.9% was determined by ICP. Pure ZnO@ZIF-8 and commercial Pt@CNT were used as controls in the catalytic experiments.

### Typical procedure for the catalytic hydrogenation of alkenes

Hydrogenation of alkenes (97% for n-hexene and 96% for cyclooctene) was carried out in ethyl acetate solution in a static hydrogen atmosphere (1 bar). In a typical experiment, the catalyst (0.01 g) was loaded in a reactor, and residual air in the reactor was expelled by flushing several times with hydrogen. Ethyl acetate (3 ml) was added in the reactor, and the mixture was sonicated for 5 min to afford a homogeneous suspension. Alkene (0.1 ml) was then added in the reactor, and the mixture was sonicated again for 5 min. After the reactor was again flushed one time with hydrogen, the reaction was allowed to proceed at 1 atm of hydrogen and 35 °C for 12 h. After the reaction, the catalyst powder was filtered off, and the filtrate was analysed by using a gas chromatograph (Agilent, 6890N) equipped with an HP-5 capillary column (Agilent) and flame ionization detector.

### Data availability

The data that support the findings of this study are available from the corresponding author upon reasonable request.

## Additional information

**How to cite this article:** Yang, Q. *et al*. Regulating the spatial distribution of metal nanoparticles within metal-organic frameworks to enhance catalytic efficiency. *Nat. Commun.* **8**, 14429 doi: 10.1038/ncomms14429 (2017).

**Publisher's note:** Springer Nature remains neutral with regard to jurisdictional claims in published maps and institutional affiliations.

## Supplementary Material

Supplementary Information Supplementary Figures, Supplementary Table, Supplementary Methods and Supplementary References
