# Supplementary material for: Regulating the spatial distribution of metal nanoparticles within metal-organic frameworks to enhance catalytic efficiency
Source: Nat Commun. 2017 Feb 14;8:14429. doi: 10.1038/ncomms14429 (PMC5316883; doi:10.1038/ncomms14429)
Supplement: Supplementary Information — Supplementary Figures, Supplementary Table, Supplementary Methods and Supplementary References [file ncomms14429-s1.pdf]

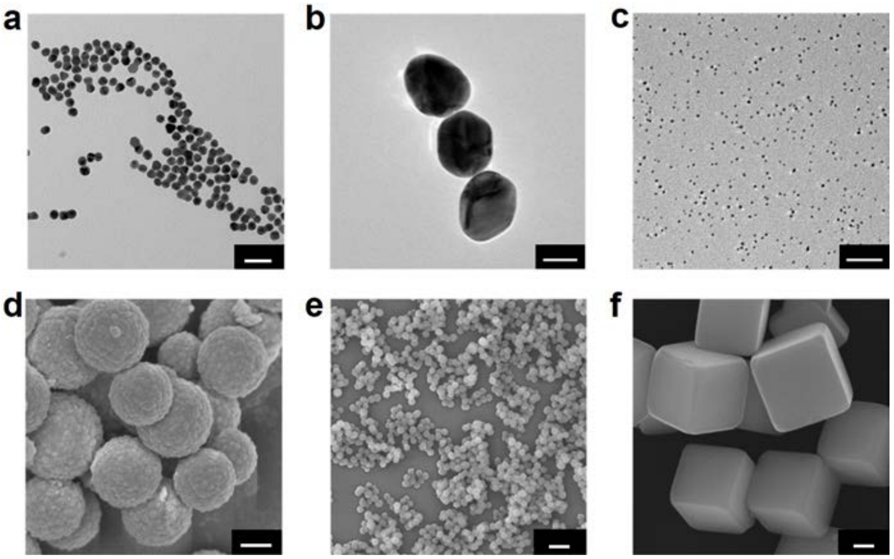

Supplementary Figure 1. SEM and TEM images of the metal nanoparticles (MNPs) and metal oxide templates. (a) 13 nm Au, (b) 60 nm Au, (c) 3.3 nm Pt, (d) ZnO spheres, (e) Al_2O_3 spheres and (f) Cu_2O cubes. Scale bar, 50 nm (a-c), 500 nm (d), 2 μm (e) and 200 nm (f).

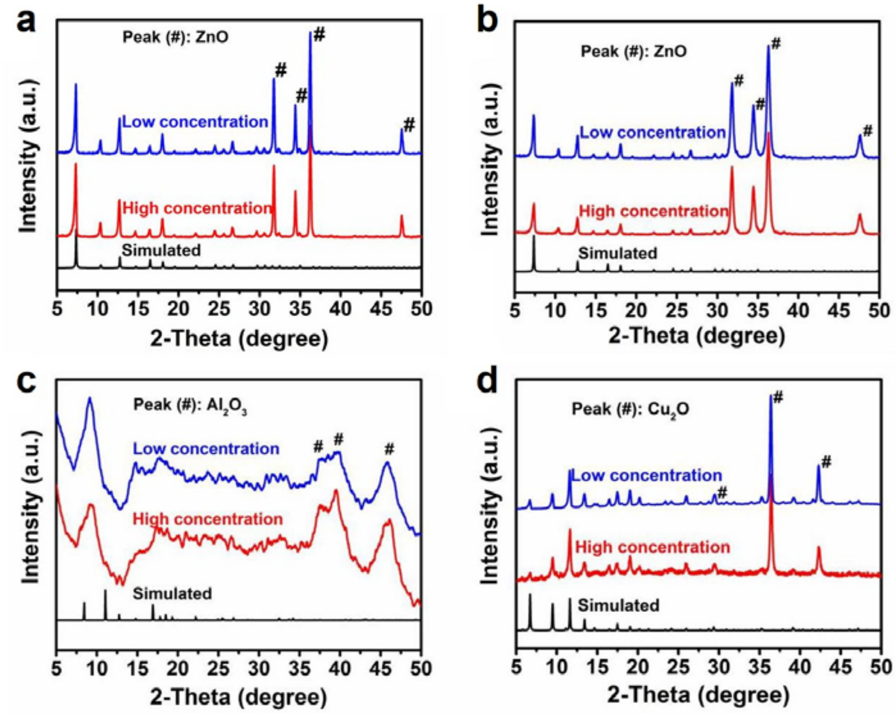

Supplementary Figure 2. XRD patterns of various types of MNPs@MOF composites: (a) 13 nm Au NPs in ZIF-8 with ZnO nanorods as template, (b) 13 nm Au NPs in ZIF-8 with ZnO spheres as template, (c) 13 nm Au NPs in HKUST-1 with Cu_2O cubes as template and (d) 3.3 nm Pt NPs in MIL-53 with Al_2O_3 spheres as template.

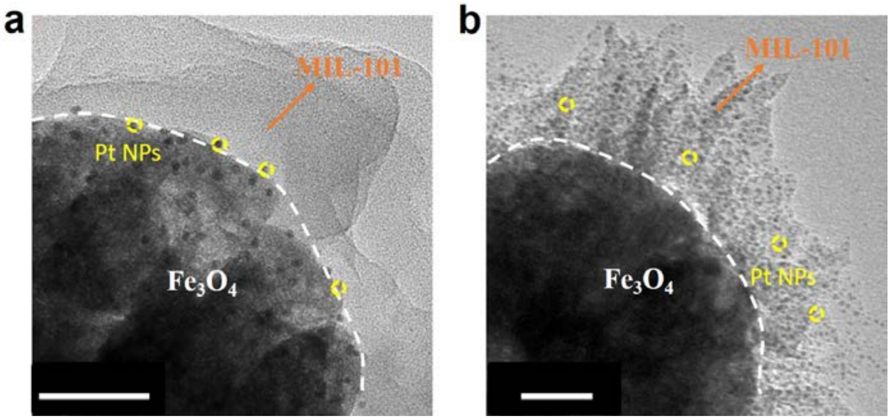

Supplementary Figure 3. The encapsulation of Pt NPs into MIL-101 with spatial regulation. TEM images of Fe_3O_4@Pt@MIL-101 composites with Pt NPs at different locations: (a) inside and (b) near to the surface of MIL-101. Scale bar, 50 nm.

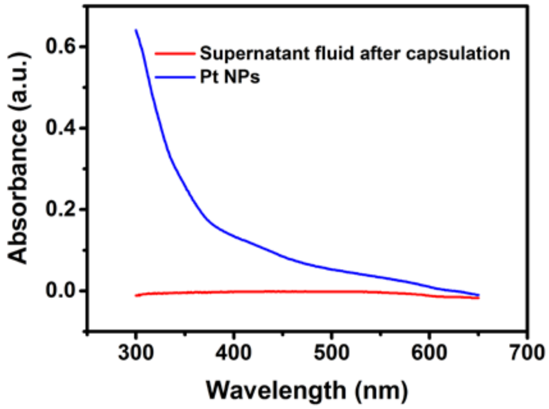

Supplementary Figure 4. UV-vis absorbance spectra of the solution after capsulation. After the capsulation, the dispersion solution was centrifuged at 6000 rpm for 3 min to remove ZnO@Pt@ZIF-8. The supernatant fluid after centrifuged shows no UV-Vis absorption peak (red line). In contrast, dispersion of Pt NPs (0.25 mg/mL) after centrifuged at 6000 rpm for 3 min shows strong UV-visible absorption (blue line). The results indicated that there is almost no loss of the nanoparticles during the capsulation.

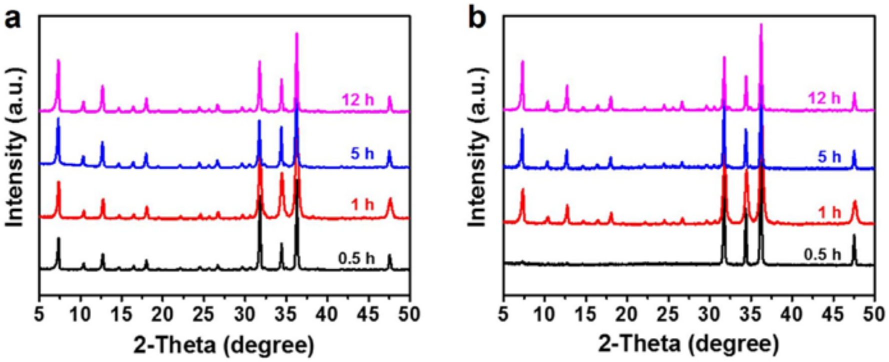

Supplementary Figure 5. XRD patterns of the ZnO NRs@Au@ZIF-8 composites at the different reaction time: (a) high concentration of Hmim; (b) low concentration of Hmim.

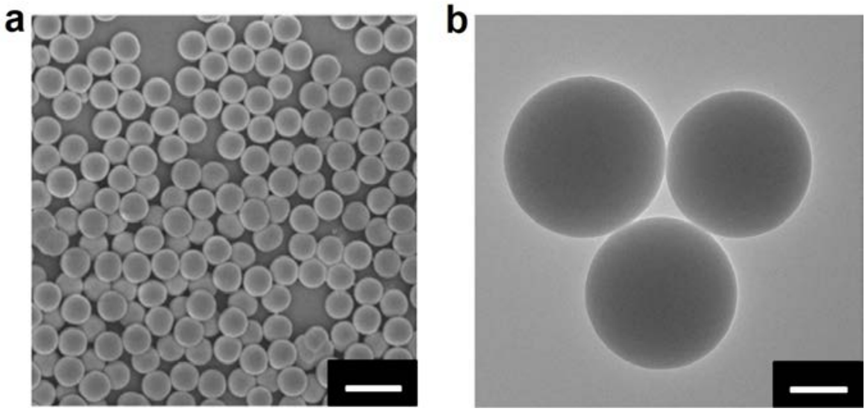

Supplementary Figure 6. (a) SEM and (b) TEM image of SiO_2 nanospheres. Scale bar, 1 μm (a) and 200 nm (b).

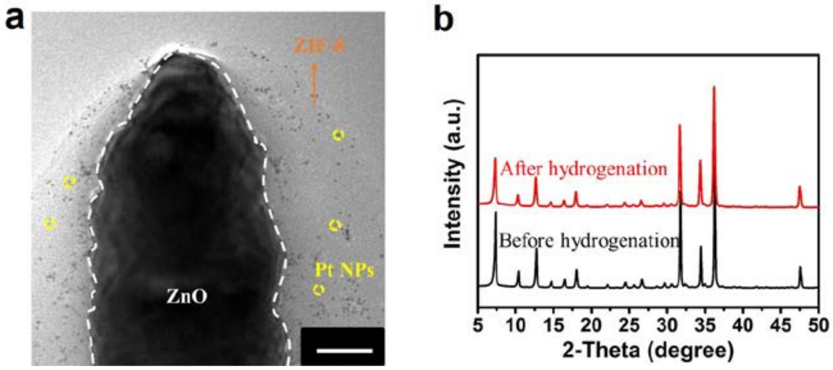

Supplementary Figure 7. (a) TEM image of Pt@ZIF-8-“sur” composite after hydrogenation of n-hexene (Scale bar, 50 nm); (b) XRD patterns of Pt@ZIF-8-“sur” composites before and after hydrogenation of n-hexene for five consecutive runs.

Supplementary Table 1. Olefin hydrogenation reactions catalyzed by ZnO@Pt@ZIF-8 composites.

**Entry**	**Catalysts**	**Time (h)**	**Conversion*** **(%)**	**CO chemisorption**** **(umol/g metal)**	**TOF***** **(h^{-1})**
	Substrate: hexene				
**1**	ZIF-8	12	0	0	0
**2**	Pt@ZIF-8-“in”	12	7	120.0	437.5
**3**	Pt@ZIF-8-“sur”	12	21	117.8	1337.3
**4**	Pt@CNT	12	60	1512.0	297.6
	Substrate: cyclooctene				
**5**	ZIF-8	12	0	0	0
**6**	Pt@ZIF-8-“in”	12	0	120.0	0
**7**	Pt@ZIF-8-“sur”	12	0	117.8	0
**8**	Pt@CNT	12	42	1512.0	198.0

*Conversion of olefins were determined by gas chromatography.

**CO chemisorption experiments were used to measure active sites of the catalysts.

***TOFs were calculated using the following equation[1]: $TOF = \frac{\text{mole number of converted olefin (mol)}}{\text{active sites (mol)} \times \text{time (h)}}$.

Supplementary Methods

Synthesis of PVP-stabilized Au NPs.[2] Au NPs were prepared by a trisodium citrate reduction method of $HAuCl_4$. For the synthesis of 13 nm Au NPs, aqueous solution of $HAuCl_4$ (0.01%, 150 mL) was added into a round bottom flask (250 mL, fitted with a reflux condenser) with rapid and continuous stirring. When the solution started to boil, an aqueous solution of trisodium citrate (1%, 4.5 mL) was added. After the mixture was refluxed for another 20 min, deep red Au NPs sol was obtained. 60 nm Au NPs were synthesized by using 13 nm Au NPs as seeds using the same procedure described above. In typical, $HAuCl_4$ (0.01%, 135 mL) was brought to a vigorous boil with rapid stirring. When the solution started to boil, the obtained 13 nm Au NPs seeds (3 mL) were added. After 2 min, an aqueous solution of trisodium citrate (1%, 0.54 mL) was added, and the mixture was refluxed for another 20 min to prepare the 60 nm Au NPs. When the Au NPs was cooled to room temperature, a solution of PVP (0.5 g, Mw = 55,000) in water (20 mL) was added dropwise into the Au NPs sol with stirring. and the mixture was further stirred at room temperature for 24 h. Finally, the PVP-stabilized Au NPs were collected by centrifugation, washed by methanol for three times, and final dispersed in methanol (absorbance at 520 nm, ~ 1).

Synthesis of PVP-stabilized Pt NPs.[3] 3.3 nm PVP-stabilized Pt NPs were prepared by refluxing a mixture of PVP (133 mg, Mw=29,000), methanol (180 mL), and aqueous solution of H_2PtCl_6 (6.0 mM, 20 mL) in a round bottom flask (500 mL) for 3 h under air. When the Pt NPs suspension was cooled to room temperature, most of the solvent was removed by rotary evaporator. The NPs in the remaining solution (~5 mL) were precipitated by acetone, and then collected by centrifugation at 6,000 rpm for 5 min. After treatment by vacuum drying for 12 h, the sample was washed with chloroform and hexane to remove excess free PVP, and finally dispersed in methanol (absorbance at 520 nm, ~ 1). .

Synthesis of ZnO nanorods.[4] 0.33 g $Zn(CH_3COO)_2·2H_2O$ and 1.2 g NaOH were separately dissolved in 4.5 mL of distilled water. Then, 10.5 mL of glycerin was dropped in the above solution. After magnetic stirring for 15 min, the mixture was transferred to a 20 mL Teflon-lined stainless-steel autoclave, and kept at 150°C for 24 h. The products were collected by centrifugation and washed several times with deionized water and ethanol. After calcination at 500 °C for 2 h in air, ZnO nanorods were obtained.

Synthesis of ZnO spheres.[5] In a typical procedure, 0.48 mmol of zinc nitrate hexahydrate ($Zn(NO_3)_2{\cdot}6H_2O$), 0.26 mmol of hexamethylenetetramine (HMT, $(CH_2)_6N_4$) and 0.12 mmol sodium citrate ($Na_3C_6H_5O_7{\cdot}2H_2O$) were dissolved into 50 mL of deionised water under stirring. The mixed aqueous solution was heated at 90 °C in water bath for 20 min and immediately washed with distilled water and ethanol several times. The ZnO spheres were produced by the calcination of zinc citrate at 600 °C for 2 h in air.

Synthesis of Cu_2O nanocubes.[6] In a typical synthesis, 0.171g $CuCl_2{\cdot}2H_2O$ dissolved into 100 mL deionized water was added into a round bottom flask with constant stirring at 55 °C. Then, 10.0 mL NaOH aqueous solution (2.0 M) was added dropwise into the above transparent light green solution. After stirring for 0.5 h, an ascorbic acid solution (10.0 mL, 0.6 M) was added dropwise into the dark brown solution. After the mixture was aged for 3 h, the product was collected by centrifugation and followed by washing with distilled water 3 times and absolute ethanol twice to remove the residual inorganic ions and polymer.

Synthesis of Al_2O_3 spheres.[7] In a typical procedure, 2.5 mM $Al_2(SO_4)_3{\cdot}16H_2O$, 7.5 mM $Al(NO_3)_3{\cdot}9H_2O$, and 100 mM urea was dissolved into 100 mL water. The reactant solutions were reacted in a 98 °C oil bath for 10 h. After the reaction, the obtained suspensions were purified from the occluded SO_4^{2-} and NO_3^- by repeated washings with deionized water. The synthesized aluminum hydrous oxide precursors were calcined at 800 °C in air to transform them into α-Al_2O_3.

Synthesis of Fe_3O_4 spheres.[8] In a typical procedure, 1.35g of $FeCl_3{\cdot}6H_2O$ was dissolved in 40 mL of ethylene glycol to form a clear solution, followed by the addition of 3.6g of sodium acetate and 1.0 g of polyethylene glycol. The mixture was stirred vigorously for 30 min and then sealed in a 50 mL Teflon lined stainless-steel autoclave. The autoclave was heated to and maintained at 200°C for 12 h, and allowed to cool to room temperature. The black products were washed 3 times with ethanol and dried at 60°C for 6 h.

Synthesis of SiO_2 nanospheres. In a typical procedure, 3 mL tetraethyl orthosilicate (TEOS, 99%) was first dissolved in a mixture of 50 mL ethanol in a glass bottle. Afterward, 30 mL of concentrated ammonia solution ($NH_3{\cdot}H_2O$, 28%) mixed with 100 mL ethanol was added to the above solution under vigorous magnetic stirring. The hydrolysis of TEOS and related condensation reactions lasted for another 8 h under continuous magnetic stirring. The SiO_2

nanospheres were washed by centrifuge with ethanol and deionized water thoroughly and finally dispersed into 50 mL water for further using.

Synthesis of metal oxide@MNPs. Metal oxide@MNPs were prepared by shaking their mixture solution overnight and washed by centrifugation.

Encapsulation of 13 nm Au NPs in MIL-53 with Al_2O_3 spheres as template. In this synthesis, Al_2O_3@13 nm-Au (~2 mg) with a certain amount of terephthalic acid (40 mg or 2 mg) was added to a glass bottle containing a mixed solvent of DMF/H_2O (4 mL, 1:1 v/v). After reacting the mixture at 60 °C for 12 h, the products were washed with DMF and ethanol three times, and Al_2O_3@Au@MIL-53 composites with core-shell structure were obtained.

Encapsulation of 13 nm Au NPs in HKUST-1 with Cu_2O cubes as template. In this synthesis, Cu_2O@13 nm-Au (~2 mg) with a certain amount of trimesic acid (20 mg or 1 mg) was added to an autoclave containing a solvent of 4 mL benzylalcohol. After reacting the mixture at 80 °C for 12 h, Cu_2O@Au@HKUST-1 composites with core-shell structure were obtained.

Encapsulation of 60 nm Au NPs in ZIF-8 with ZnO spheres as template. In a typical experiment, ZnO@60 nm-Au (~10 mg) with a certain amount of Hmim (2 mmol or 0.64 mmol) was added to a glass bottle containing a mixed solvent of DMF/H2O (16 mL, 3:1 v/v). After reacting the mixture at 70 °C for 12 h, the products were washed by ethanol three times, and ZnO@Au@ZIF-8 composites with core-shell structure were obtained.

Encapsulation of 3.3 nm Pt NPs in MIL-101 with Fe_3O_4 spheres as template. In this synthesis, Fe_3O_4@3.3 nm-Pt (~2 mg) with a certain amount of terephthalic acid (40 mg or 2 mg) was added to an autoclave containing a mixed solvent of DMF/H_2O (12 mL, 1:3 v/v).. After reacting the mixture at 90 °C for 12 h, Fe_3O_4@Pt@MIL-101 composites with core-shell structure were obtained.

Encapsulation of 3.3 nm Pt NPs in MIL-53 with Al_2O_3 spheres as template. In this synthesis, Al_2O_3@3.3nm-Pt (~2 mg) with a certain amount of terephthalic acid (40 mg or 2 mg) was added to a glass bottle containing a mixed solvent of DMF/H_2O (4 mL, 1:1 v/v). After reacting the mixture at 60 °C for 12 h, the products were washed with DMF and ethanol three times, and Al_2O_3@Pt@MIL-53 composites with core-shell structure were obtained.

Supplementary References

1 Li, Z. *et al.* Platinum-nickel frame within metal-organic framework fabricated in situ for hydrogen enrichment and molecular sieving. *Nat. Commun.* **6**, 8248 (2015).

2 Frens, G. Controlled nucleation for the regulation of the particle size in monodisperse gold suspensions. *Nature* **241**, 20-22 (1973).

3 Teranishi, T., Hosoe, M., Tanaka, T. & Miyake, M. Size control of monodispersed Pt nanoparticles and their 2D organization by electrophoretic deposition. *J. Phys. Chem. B* **103**, 3818-3827 (1999).

4 Han, X.-G. *et al.* Control of the surface of ZnO nanostructures by selective wet-chemical etching. *J. Phys. Chem. C* **114**, 10114-10118 (2010).

5 Xie, Q., Li, J., Tian, Q. & Shi, R. Template-free synthesis of zinc citrate yolk–shell microspheres and their transformation to ZnO yolk–shell nanospheres. *J. Mater. Chem.* **22**, 13541-13547 (2012).

6 Zhang, D.-F. *et al.* Delicate control of crystallographic facet-oriented Cu_2O nanocrystals and the correlated adsorption ability. *J. Mater. Chem.* **19**, 5220-5225 (2009).

7 Roh, H.-S. *et al.* Size-controlled synthesis of monodispersed mesoporous α-Alumina spheres by a template-free forced hydrolysis method. *Dalton Trans.* **40**, 6901-6905 (2011).

8 Deng, H. *et al.* Monodisperse magnetic single-crystal ferrite microspheres. *Angew. Chem. Int. Ed.* **44**, 2782-2785 (2005).
